# A Generalized Spatial Modulation System Using Massive MIMO Space Time Coding Antenna Grouping

**DOI:** 10.3390/e22121350

**Published:** 2020-11-30

**Authors:** Amira I. Zaki, Mahmoud Nassar, Moustafa H. Aly, Waleed K. Badawi

**Affiliations:** College of Engineering and Technology, Arab Academy for Science, Technology and Maritime Transport, Alexandria 1029, Egypt; amzak10@aast.edu (A.I.Z.); engmmnassar@gmail.com (M.N.); waleedbadawi@aast.edu (W.K.B.)

**Keywords:** space time coding, spatial modulation, generalized spatial modulation, multiple input multiple output

## Abstract

Massive multiple input multiple output (MIMO), also known as a very large-scale MIMO, is an emerging technology in wireless communications that increases capacity compared to MIMO systems. The massive MIMO communication technique is currently forming a major part of ongoing research. The main issue for massive MIMO improvements depends on the number of transmitting antennas to increase the data rate and minimize bit error rate (BER). To enhance the data rate and BER, new coding and modulation techniques are required. In this paper, a generalized spatial modulation (GSM) with antenna grouping space time coding technique (STC) is proposed. The proposed GSM-STC technique is based on space time coding of two successive GSM-modulated data symbols on two subgroups of antennas to improve data rate and to minimize BER. Moreover, the proposed GSM-STC system can offer spatial diversity gains and can also increase the reliability of the wireless channel by providing replicas of the received signal. The simulation results show that GSM-STC achieves better performance compared to conventional GSM techniques in terms of data rate and BER, leading to good potential for massive MIMO by using subgroups of antennas.

## 1. Introduction

In modern communication systems, network traffic and the demand for a greater data rates are growing rapidly due to the rapid increase of using smart devices and cloud computing. Due to users increasing demand for high data rates all over the world, a lot of research is being done to achieve their requirements. The multiple input multiple output (MIMO) techniques are highly recommended, as they can achieve higher throughput compared to the single input single output systems (SISO) [1]. Increasing the number of transmitting antennas has the ability to enhance system performance due to introducing a diversity gain in the received data symbols which is enhanced by increasing the number of transmit antennas [2]. The MIMO system can also reduce the effect of channel on the information data sent from a transmitter to a receiver end [3]. On the other hand, the MIMO system suffers from high complexity during implementation. The MIMO wireless communication system is overviewed in [4], which shows that the new massive MIMO system is an emerging base station (BS) technology that deploys a very large number of BS antennas to enhance spectral efficiency, communication reliability and increase the transmission data rate. Another approach to enhanced data rate is using beamforming (BF) to perform transmit diversity and enhance the average BER (bit error rate), especially in correlated fading channels, but this still does not achieve the nowadays required high data rates [5]. The Massive MIMO reduces the inter user interface and enhances the physical security due to transmission over orthogonal MIMO channels [6]. This essential tradeoff between the diversity gain and the multiplexing gain is critical to the performance of MIMO systems [7]. So, another technique has to be proposed. A lot of research has been done to enhance the performance of a massive MIMO system.

In order to increase the spectral efficiency and to reduce the inter-channel interference (ICI), a spatial multiplexing MIMO technique is proposed called spatial modulation (SM) [8]. In general, SM using MIMO can achieve rate improvement and thus higher spectral efficiency. However, one of the chronic problems is that it gives a higher BER when fixing the signal-to-noise ratio (SNR). A specified procedure is performed in [9] to enhance the BER by activating only one antenna at the transmitter side at each instance to transmit a certain data symbol. In order to increase the overall spectral efficiency, the base–two logarithm of the number of transmit antennas is used by S. Buzzi et al. [10] using an active antenna index. Furthermore, the data sent depends on the incoming random data bits, taking into consideration that the total number of transmit antennas must be a power of two. At the receiver side, a detector should be added that can jointly estimate the active antenna index and the sent data symbol [11]. In [12], the proposed SM decoder is performed. Then, in [13], an SM combined with trellis-coded modulation (TCM) was recently proposed.

Another approach is done in [14] by using antennas grouping at the transmitter with SM to increase the data rate by setting antennas in more than one group and antennas in the same group to be planned at equal distances. Recently, when the spatial dimension was introduced, a capacity gain could be achieved. It is coordinated by the antenna indices to be introduced for the developed MIMO schemes.

In order to avoid the ICI and the inter antenna interference (IAI), only one antenna must be active at transmitter, which results in a further reduction in decoding complexity and flexibility in system design [15]. Furthermore, a novel scheme approaching even higher capacity by combining the amplitude/phase modulation techniques with antenna index modulation, named multiple access spatial modulation (SM), is proposed by Z. Bouida et al. in [16] in order to expand the constellation symbols for a three dimensional one (the spatial dimension and the complex plane are included).

Symbols are emitted through the active antennas through a conventional modulator. Therefore, the information is transferred not only by the amplitude/phase modulation techniques, but also by the antenna indices [17]. The SM and space shift key (SSK) modulation systems can eliminate ICI and IAI by using only one active antenna all over the transmission. This will limit the utilization of spatial dimension and design flexibility. Hence, a low complexity decoder is enabled for prominent performance. Another approach is performed in [18] for multiple active transmit antennas in SM (MA-SM) by adding a phase to each transmitted symbol to enhance the BER and achieve a high data rate. At the receiver, antenna detector used to detect the activated combination of the antenna group at transmitter. However, this system suffers from high BER when increasing the transmit antennas and suffers from complexity.

Another approach is performed in [19] for multiple active transmit antennas in SM (MA-SM) by adding a phase for each transmitted symbol to enhance the BER and achieve a high data rate. At the receiver, an antenna detector is used to estimate the activated set of antenna combinations at the receiver. However, this system suffers from high BER when increasing the transmit antennas and suffers from complexity.

Another way to determine the number of transmit antennas is proposed by N. Fernando in [20] to reduce system complexity. In the generalized spatial modulation (GSM), a set of transmit antennas are to be activated at the same time to simultaneously transmit a data symbol. In general, the transmitted information in GSM is conveyed over the active set of antennas at the transmitter and the transmitted symbol from a signal constellation. The number of antennas needed at the transmitter required to match a certain spectral efficiency in GSM is equal to half number of transmit antennas in SM, and a generalized space shift keying modulation (GSSK) is proposed in [21].

One of the key advantages of GSM is that it can transmit the same data symbol from more than one antenna at a time, which will overcome the effects of ICI at the receiver. GSM also achieves spatial diversity gains and increases the reliability of the wireless channel by providing replicas of the transmitted signal to the receiver. In order to avoid inter-symbol interference (ISI), the activated transmit antennas must be synchronized. The maximum likelihood (ML) detection algorithm is taken into consideration at the receiver in order to estimate the activated set of antenna combinations at the transmitter and the transmitted constellation symbol.

To achieve a high transmit rate, GSM requires a large number of transmit antennas that increases the complexity of the system exponentially. Moreover, GSM decoders suffer from high complexity and the linear decoder also suffers from poor performance, so a new technique is to be proposed.

In this paper, a new technique is proposed based on GSM modulation and space time encoding of the GSM-modulated symbols on different antenna subgroups. In the proposed technique, STC is performed for two successive GSM modulated symbols on two antenna subgroups. The STC is used to provide transmit diversity for the multiple antenna fading channels. Antenna grouping is utilized to increase the data rate and decrease the BER. The proposed technique (GSM-STC) uses the ML detection algorithm at the receiver to assess the set of activated combination of transmit antennas and the transmitted constellation symbols. The proposed technique is expected to offer a high data rate with low BER. The proposed technique is evaluated experimentally considering selective fading channels for different types of modulation. The simulation results show that the proposed technique achieves a higher data rate and lower BER compared to the conventional and the modified GSM techniques.

The remainder of the paper is organized as follows. Section 2 presents the GSM system model with a new modification by increasing antenna number and using Quadrature Amplitude Modulation (QAM) modulation. Then, the GSM-STC is presented using two subgroups of antennas and with QAM modulation. The simulation results for the modified GSM and GSM-STC technique are displayed and discussed in Section 3. This is followed by the main conclusions in Section 4, showing the advantages of the proposed system.

## 2. System Model and Analysis

In this section, the GSM system model will be presented using one group of antennas and selective fading channel in A. The GSM-STC will be presented in B using two subgroups of antennas and selective fading channel.

### 2.1. GSM System Model

**Figure 1:**
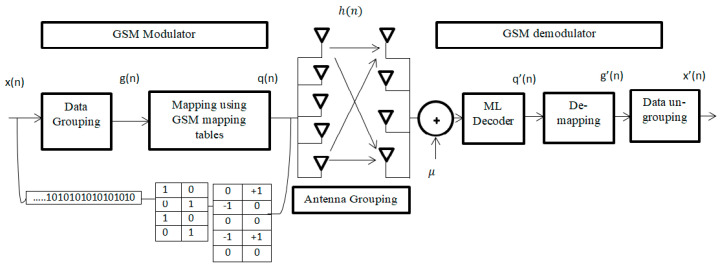
GSM (generalized spatial modulation) system model.

One of the great challenges for massive MIMO to achieve a high data rate. The GSM system model can achieve a high data rate by using a set of transmit antennas to send the same constellation symbol. The proposed system is explained in Figure 1, showing that a group of antennas is formed and uses the same constellation point. $N_{t}$ refers to the total number of antennas available at transmitter and $N_{u}$ is the number of active transmit antennas. However, the set of antenna combinations that will be used for transmission must be a power of two.

The proposed GSM system model is designed for $N_{t}$ = 5 and $N_{u}$ = 2.

Thus, the possible antenna combinations $N_{c}$ = $2^{ml}$, $m_{l}$ = $[\log_{2}\begin{array}{c} N_{t}\\ N_{u} \end{array}$]

The mapping data below are considered in [22] to map the incoming data bits to a spatial symbol and a data symbol, which is used before for Quadrature Phase Shift Keying (QPSK) as illustrated in Table 1.

In the example of the grouped bits g(n) = [0 0 0 0], the first three bits 000 refer to the antenna combination (1,2) and the rest bit 0 refers to the transmitted symbol −1. So, if g(n) = [1 0 1 1], the antenna combination will be (2,4) and the transmitted symbol will be +1.

The GSM model is illustrated in Figure 1. The first ml bits are mapped to be used for the set of antenna combinations and the set of the remaining bits ms are modulated using M-QAM modulation, where M = $2^{ms}.$

In general, the total number of bits that will be transmitted using the GSM is given by [22]:

(1) $$m=ms+ml=\log_{2}(N_{t}/N_{u})+\log_{2}M$$

The modulated signal for GSM is transmitted over a MIMO selective Rayleigh fading channel, H. The channel matrix H has the dimension size of $N_{r}\times N_{t}$, where $N_{r}$ and $N_{t}$ are, respectively, the number of the receiving and transmitting antennas.

The input data stream q(n) is grouped firstly using data grouping and then the output g(n) is mapped using GSM mapping (Table 1).

The received signal at any given time

(2) $$Y=h^{\prime }{}_{l,s}+\mu$$

where s is the symbol transmitted through the set of antenna combination, s∈ M-QAM l = $\left[l_{1},\;l_{2},\;\ldots \ldots .,\;l_{Nu}\right]\in \emptyset$, $l_{n}$ refers to the index of $n^{th}$ antenna in the set of antennas, l and ∅ refer to the set of antenna combination, and μ is an Additive White Gaussian Noise (AWGN) vector with zero–mean.

At the receiver, the ML algorithm is used to decode the spatial symbol and the data symbol as follows

(3) $$\left[l,s\right]=\arg\;\min\overset{Nr}{\underset{i=1}{\sum }}{\left|y_{i}-h_{l,i}s\right|}^{2}$$

where

(4) $$Py\langle Y|s,l,H\rangle =\frac{1}{\left(\pi \sigma^{2}\right)}exp\left(-\frac{〚Y-h_{l\;s\;}{〛}^{2}}{\sigma^{2}}\right)$$

Here, Py〈Y|s,l,H〉 is the probability density function (PDF) of *Y* conditional on [l,s] and *H*, and $〚{〛}^{2}$ is the Frobenius norm [22].

### 2.2. GSM-STC Model

**Figure 2:**
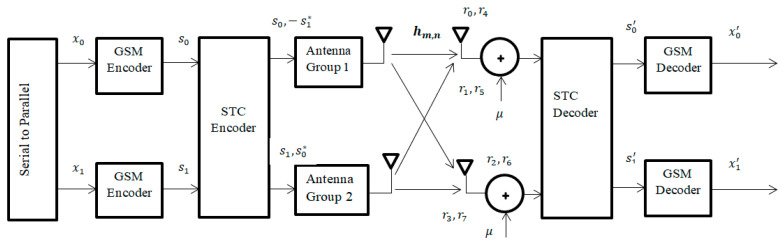
GSM-STC (space time coding technique) system model.

In order to achieve a high data rate and diversity, GSM-STC is designed. The GSM-STC will use two subgroups of antennas at the transmitter using the combination method [23] to achieve diversity with a high data rate and low bit error rate. Two groups will be added in the transmitter and each group has $N_{t}$ total transmitting antenna and $N_{u}$ number of active antennas per group. The transmitting symbols will be during two time slots, t and t+τ, as seen in Figure 2.

In this system, two subgroups of antennas are proposed at the transmitter, and each group has $N_{t}$ antenna and $N_{u}$ active antennas. At the receiver, we have $N_{r}$ receiving antennas with the same number of active antennas at transmitter.

Let $S={\left[s_{0},s_{1}\right]}^{T}$ be the un-coded M-QAM information symbol vector that will be transmitted over two sequential OFDM symbols, so the Alamouti scheme generates the following code word matrix:

(5) $$X=\begin{array}{cc} s_{0} & -s_{1}^{*}\\ s_{1} & s_{0}^{*} \end{array}$$

The subgroup antennas are illustrated in Figure 2. At time t, the first group will transmit symbol $s_{0}$ and the second group will transmit symbol $s_{1}$. At time (t+τ), the first group will transmit symbol $-s_{1}^{*}$ and the second group will transmit symbol $s_{0}^{*}$.

At the transmitted time t, active antennas from first group send $s_{0}$ symbols and $s_{1}$ will be transmitted from second group at same time. At time +τ, active antennas from first group send $-s1^{*}$ symbols and $s0^{*}$ will be transmitted from second group at same time too. Table 2 shows that the encoding and transmission sequence of the information symbols for this configuration is identical to the case single receiver. In Table 2, $h_{m,n}$ indicates the channel between transmitter and receiver, where n is the number of Rx antenna, n = 1, 2, 3, 4, and m is the number of Tx antenna, m = 1, 2, 3… 10.

$r_{0},r_{1},\;r_{2},\;r_{3},\;r_{4},\;r_{5},\;r_{6}\;and\;r_{7}$ are the complex random variables at the receiver, including noise and interference. The following two signals are built from a combiner that will be sent to the ML decoder as

(6) $$s_{0}=h_{0}^{*}r_{0}+h_{1}r_{1}^{*}+h_{2}^{*}r_{2}+h_{3}r_{3}^{*}+h_{4}^{*}r_{4}+h_{5}r_{5}^{*}+h_{6}^{*}r_{6}+h_{7}r_{7}^{*}$$

(7) $$s_{1}=h_{1}^{*}r_{0}-h_{0}r_{1}^{*}+h_{3}^{*}r_{2}-h_{2}r_{3}^{*}+h_{5}^{*}r_{4}-h_{4}r_{5}^{*}+h_{7}^{*}r_{6}-h_{6}r_{7}^{*}$$

Therefore, the resulting diversity order from the new two-branch transmit diversity scheme with two receivers is equal to that of the four-branch multi-rate resource control (MRRC) scheme. It is important to highlight that the combined signals from the two receive antennas and the combined signals from each receive antenna are simply added, i.e., the combining scheme is more identical to the case with a single receive antenna. One can draw a concern that, if we use two transmit and M receive antennas, one of the transmitting antennas can use the combiner for each receive antenna and then easily add the combined signals from all the receive antennas to obtain the same diversity order as 2M-branch MRRC. Finally, when using two antennas at the transmitter side, the scheme doubles the diversity order of systems with one transmit and multiple receive antennas.

## 3. Simulation Results

In the following, simulation results for the proposed GSM-STC technique are compared to conventional GSM [22] and modified GSM. A set of trials were done in terms of modulation techniques, the number of subcarriers and channel taps. These trials are discussed in the following subsections. The simulation results are displayed and discussed. The selective fading channel and QAM modulation will be used.

The proposed GSM-STC is evaluated and compared with the conventional and the modified GSM. The simulation test is done for conventional GSM by using one group of antennas containing five antennas at the transmitter. Only two of them are active for each data symbol. At the receiver side, two antennas are used. The modified GSM is examined through simulation for the same number of antennas at the transmitter, like the conventional GSM. However, at the receiver side, four antennas are used. The proposed GSM-STC is implemented using two groups of antennas at the transmitter, where each group has five antennas, with only two of them active per group for each data symbol. A selective Rayleigh fading channel is used with QAM modulation schemes.

The simulation parameters used in the evaluation are summarized in Table 3.

**Figure 3:**
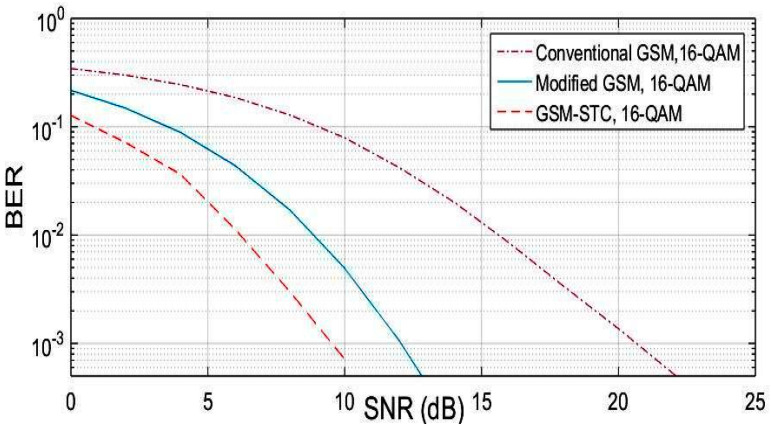
BER (bit error rate) comparison for the proposed technique (GSM-STC) with GSM techniques versus SNR (signal-to-noise ratio) using 16-QAM.

The BER performance versus SNR for M=16 and selective fading channel is shown in Figure 3. The comparison is done for the BER performance of conventional GSM. Simulation results show that the proposed technique outperforms the conventional GSM techniques. The SNR is enhanced by 11 dB as compared to conventional GSM and by 4 dB as compared to modified GSM.

**Figure 4:**
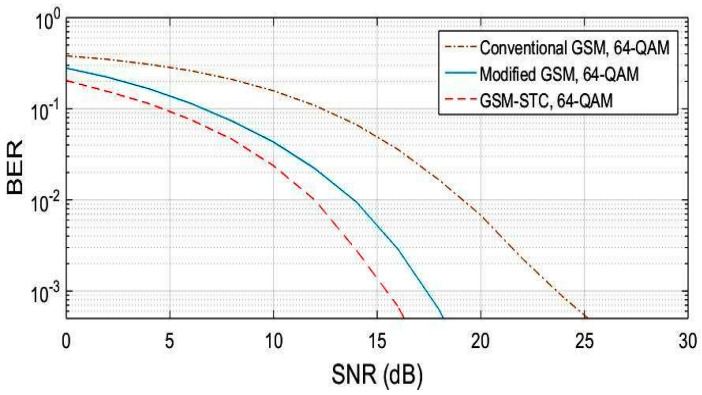
BER comparison for the proposed technique (GSM-STC) with GSM techniques versus SNR using 64-QAM.

**Figure 5:**
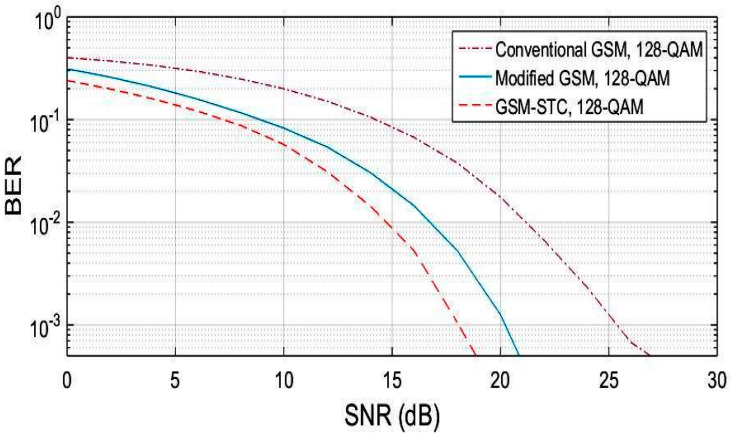
BER comparison for the proposed technique (GSM-STC) with GSM techniques versus SNR using 128-QAM.

**Figure 6:**
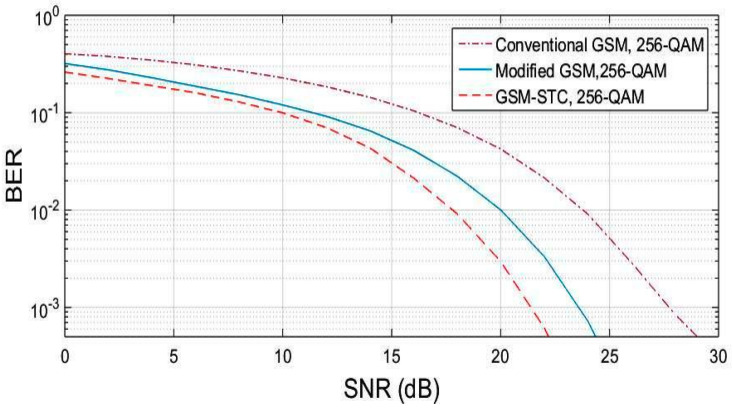
BER comparison for the proposed technique (GSM-STC) with GSM techniques versus SNR using 256-QAM.

The procedure is repeated for M = 64, M = 128, M = 256. The obtained results are displayed in Figure 4, Figure 5 and Figure 6, for the proposed system (GSM-STC) in comparison with conventional and modified GSM. The preference for STC-GSM versus conventional GSM and modified GSM is illustrated in Table 4, which summarizes the obtained SNR in a comparative form.

**Figure 7:**
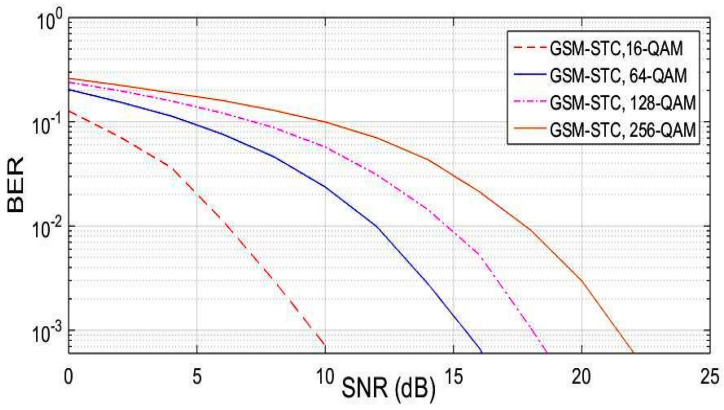
BER versus SNR of the proposed technique (GSM-STC) in comparison for a different modulation technique.

The GSM-STC technique has a better performance and matches massive MIMO requirements due to the ability to increase the data rate by the use of two subgroups of antennas at the transmitter to achieve diversity and enhance BER. The comparison between QAM modulation schemes with the same number of antennas at transmitter $N_{t}$ = 10 and same active antennas per group $N_{u}$ = 2, and also with the same number of receiving antennas $N_{r}$ = 4, is shown in Figure 7. This reveals that the 16-QAM is better than all the others.

## 4. Conclusions

In this paper, a new technique is proposed to match 5G requirements. The proposed technique is based on using STC and antenna grouping. The use of STC and antenna grouping enhances the performance of the proposed technique in terms of data rate and BER. On the other hand, the conventional GSM technique is modified by changing the number of transmitting and receiving antennas, considering a selective fading channel and QAM modulation. The proposed technique and the modified GSM are evaluated, and the simulation results show that the GSM-STC outperforms the conventional and modified GSM. The simulation results reveal that the SNR for the proposed technique is enhanced by 11 dB and 4 dB compared to conventional and modified GSM, respectively, for 16-QAM. This means that GSM-STC is a good candidate for massive MIMO due to its advantage in achieving diversity gain and increased data rate with lower BER. Moreover, the simulation results also show that the modified GSM technique achieves a higher data rate compared to the conventional GSM technique.

## Figures and Tables

**Table 1:** GSM mapping table for $N_{t}=5$, $N_{u}=2$ [22].

Group of Bits	Set of Antennas (l)	Transmitted Symbol (s)
0001	(1,2)	+1
0000	(1,2)	−1
0011	(1,3)	+1
0010	(1,3)	−1
0101	(1,4)	+1
0100	(1,4)	−1
0111	(1,5)	+1
0110	(1,5)	−1
1001	(2,3)	+1
1000	(2,3)	−1
1011	(2,4)	+1
1010	(2,4)	−1
1101	(3,5)	+1
1100	(3,5)	−1
1111	(4,5)	+1
1110	(4,5)	−1

**Table 2:** The received signal using two antennas at receiver.

	Receiving Antenna 1	Receiving Antenna 2
Time t	$\begin{array}{c} r_{0}=h_{0}s_{0}+h_{1}s_{1}+\mu_{0}\\ r_{4}=h_{4}s_{0}+h_{5}s_{1}+\mu_{4} \end{array}$	$\begin{array}{c} r_{2}=h_{2}s_{0}+h_{3}s_{1}+\mu_{3}\\ r_{6}=h_{6}s_{0}+h_{7}s_{1}+\mu_{6} \end{array}$
Time t+τ	$\begin{array}{c} r_{1}=-h_{0}s_{1}^{*}+h_{1}s_{0}^{*}+\mu_{1}\\ r_{5}=-h_{4}s_{1}^{*}+h_{5}s_{0}^{*}+\mu_{5} \end{array}$	$\begin{array}{c} r_{3}=-h_{2}s_{1}^{*}+h_{3}s_{0}^{*}+\mu_{3}\\ r_{7}=-h_{6}s_{1}^{*}+h_{7}s_{0}^{*}+\mu_{7} \end{array}$

**Table 3:** Simulation parameters.

Types	Conventional GSM	Modified GSM	GSM-STC
Number of antenna groups at transmitter	One group	One group	Two groups
Transmitting antennas $N_{t}$	5	5	10
Active antennas at transmitter $N_{u}$	2	2	4
Receiving antennas $N_{r}$	2	4	4

**Table 4:** SNR comparison between GSM-STC, modified GSM and conventional GSM.

Model	16-QAM	64-QAM	128-QAM	256-QAM
STC-GSM with conventional GSM	11 dB	10 dB	9 dB	8 dB
STC-GSM with modified GSM	4 dB	3 dB	2 dB	1 dB
